# Severe Adenovirus Pneumonia Requiring Extracorporeal Membrane Oxygenation Support in Immunocompetent Children

**DOI:** 10.3389/fped.2020.00162

**Published:** 2020-04-15

**Authors:** Xuefei Chen, Jianhai Lv, Lu Qin, Chaochun Zou, Lanfang Tang

**Affiliations:** ^1^Department of Endocrinology, The Children's Hospital, Zhejiang University School of Medicine, National Clinical Research Center for Child Health, Hangzhou, China; ^2^Department of Pulmonology, The Children's Hospital, Zhejiang University School of Medicine, National Clinical Research Center for Child Health, Hangzhou, China; ^3^Department of Pediatrics, Shangyu People's Hospital, Shaoxing, China

**Keywords:** adenovirus pneumonia, extracorporeal membrane oxygenation, acute respiratory distress syndrome, survival rate, risk factors

## Abstract

**Objective:** To highlight severe adenovirus pneumonia in immunocompetent patients by analysis of severe adenovirus pneumonia associated with acute respiratory distress syndrome in whom extracorporeal membrane oxygenation (ECMO) support is required.

**Methods:**Pediatric patients with adenovirus pneumonia and ECMO supports in our hospital from February 2018 to May 2019 were retrospectively analyzed, and having 100 common adenovirus pneumonia children as a control.

**Results:**A total of 8 patients, including 4 boys (50.0%), were enrolled. They were previously immunocompetent with a median age of 31 months. They were admitted as persistent fever and cough for more than one week. Median time prior to development of respiratory failure requiring intubation and invasive mechanical ventilation was 5 days. Venoarterial ECMO support as rescue ventilation was instituted after a median time of 24.5 h of conventional mechanical ventilator support. The median duration on ECMO support was 9 days and mechanical ventilation was 14 days, respectively. Six patients (75%) were recovered and 2 (25%) died. Median length of stay in ICU and hospital were 27.5 days and 47.5 days, respectively.

**Conclusion:**The promising outcomes of our cases suggested that ECMO support for rescue ventilation may be considered when symptoms deteriorated in adenovirus pneumonia patients, and may improve outcome. However, sequelae of adenovirus pneumonia and ECMO-related complications should also be taken into account.

## Introduction

Human adenoviruses (HAdVs) are non-enveloped DNA viruses associated with a wide range of clinical manifestations (1). HAdVs infections occur primarily in children younger than 5 years of age and account for 2–5% of all pediatric respiratory illnesses and 4–10% of childhood pneumonias (2, 3). In China, HAdVs have caused respiratory tract infections outbreaks and several cases of severe pneumonia (4) in Beijing (5–7) and other regions (8–11), which has raised concerns. A retrospective study of adenovirus in the 175 autopsied pulmonary tissues of pediatric fatal pneumonia in South China showed the positive percentage of adenovirus was 9.14% (11). To date, at least 67 immunologically distinct serotypes of HAdVs have been recognized and classified into 7 subgroups (A-G) based on hemagglutinin properties, DNA homology and biochemical characteristics (12). HAdVs serotypes 3, 7, 21 and 55 appear to be most commonly associated with severe lower respiratory tract infections in children (13–15).

Adenovirus pneumonia was speculated to occur in two phases (16). Acute extensive pulmonary consolidation mimicking the bacteria pneumonia and accompanying systemic compromise characterized by multi-organ damage, caused significant morbidity and considerable mortality (15, 17). A chronic phase characterized by persistent wheezing and crackles was followed in some cases, sometimes severe enough to require mechanical ventilation support or extracorporeal life support, and development of bronchiectasis, necrotizing pneumonia, bronchiolitis obliterans (BO), atelectasis, and cor pulmonale (18–21). It can incite intensive and continuous infection with the utilization of a detective or compromised immune system sometimes resulting in lethality (1). Life-threatening adenovirus pneumonia has previously been described in immunocompromised patients (22, 23), but was relatively rare in immunocompetent individuals (1, 16). Extracorporeal membrane oxygenation (ECMO) was first introduced in 1970 as a means of cardiopulmonary support for patients with potentially reversible cardiac and/or respiratory failure in whom maximal conventional ventilator strategies have been exhausted (24). The successful use of ECMO in patients with severe hypoxemic respiratory failure during the influenza pandemic (25, 26) has increased the practice of this salvage therapy over the past decade (27). However, ECMO used to support life-threatening adenovirus pneumonia was rarely reported. Here, we report 8 cases of severe adenovirus pneumonia associated with acute respiratory distress syndrome (ARDS) in whom conventional mechanical ventilation failed and required ECMO support to highlight this rare condition, and set a comparison group to reveal the risk factors of severe adenovirus pneumonia requiring ECMO support.

## Materials and Methods

### Objectives

A total of 8 patients admitted to our unit with severe adenovirus pneumonia requiring ECMO support from February 2018 to May 2019 were retrospectively analyzed. Clinical data, including demographic characteristics (age and gender), clinical symptoms and signs, laboratory data, microbiological findings, imaging examinations, complications, treatments, and outcomes, were collected and analyzed. A random group of 100 children extracted from a total of 763 patients with common adenovirus pneumonia hospitalized during the same period was involved as a control. The 100 children were all previously healthy and the one who required ECMO support was excluded. The requirement for informed consent by individual patients was waived given the retrospective nature of the study.

### Diagnostical Methods

Nasopharyngeal swab samples, sputum, bronchoalveolar lavage fluid (BALF), pleural fluid samples and/or serum were collected for microbiological tests including immunofluorescence staining, antigen and antibody tests, nucleic acid detection and bacterial culture. Pathogen high-throughput genome sequencing (BGI, Inc, China) of BALF was performed in 3 patients to screen for bacteria, viruses, fungi, parasites, mycobacterium tuberculosis complex, mycoplasma, and chlamydia.

Adenovirus pneumonia was diagnosed based on the presence of adenovirus detected in BALF, pleural fluid or nasopharyngeal swab samples by immunofluorescence microscopy, measurement of antibodies in paired serum samples or molecular methods, and chest radiographic changes combined with the presence of attributable symptoms and signs. ARDS was diagnosed according to the consensus recommendations from the Pediatric Acute Lung Injury Consensus Conference (28), and mechanical ventilation would be considered early in children at risk for ARDS. There was no worldwide consensus for definite parameters or clinical conditions that ECMO should be initiated. In our unit, ECMO will be considered in respiratory failure not responding to conventional ventilator and pharmacologic therapies.

### Statistical Analyses

Measurement variables were summarized using median or mean and ranges, and enumeration variables using frequencies and percentages. Student's *t*-test or the Mann-Whitney *U*-test was used to compare measurement variables. Chi-square test was used to test for enumeration variables. A *p* < 0.05 was considered significant. Statistical analysis was performed by SPSS software, version 20.0.

## Results

### Clinical and Laboratory Data

All 8 patients were previously healthy immunocompetent children without any underlying diseases. They were 4 boys (50.0%) and 4 girls with a median age of 31 months (range, 13 months to 61 months). Except one patient was born at 35 weeks due to premature rupture of membranes, the others were born at term. All individuals were born by vaginal delivery without history of asphyxia at birth.

Fever and cough over one week were presented in all 8 patients with a mean maximum body temperature of 40.2°C (range, 39.8–40.9°C) at the onset of illness. All patients had tachypnea and 3 had wheeze when admitted to hospital. Rales and “three concave sign” were noted in 5 patients. Decreased breath sounds were observed in 2 patients. Neurological manifestations, such as drowsiness, were observed in 2 patients while positive Babinski sign in one. White blood cell counts of them were low or within the normal rage while lymphopenia occurred in 6 and thrombocytopenia in 2 patients. C-reactive protein and procalcitonin levels were elevated in 6 and 7 patients with the median value of 53.0 μg/L and 4.5 μg/L, respectively. Elevated lactate dehydrogenase was found in all 8 patients with the median value of 1458.5 U/L, and 4 patients over 1,000 U/L. Markedly raised levels (greater than twice the normal) of alanine aminotransferase, creatinine, and creatine kinase-MB were observed in one, one and 2 patients with the maximum value of 171, 169, and 161 U/L, respectively (Table 1).

**Table 1:** Demographic, clinical characteristics and laboratory data of patients with adenovirus pneumonia on the first day of admission.

**No**	Age^*^/Sex	**Symptoms, signs**	**T_**max**_ (^**°**^C)**	**Breath rate (/min)**	**WBC (10^**9**^/L)**	**Neutrophil (%)**	**Lymphocyte (%)**	**CRP (mg/L)**	**PCT (ng/ml)**	**Hemoglobin (g/L)**	**Platelet (10^**9**^/L)**	**ALT (U/L)**	**Cr (umol/L)**	**CK-MB (U/L)**	**LDH (U/L)**
1	49/F	Cough, fever, diarrhea	40	36	12.08	85.4	13.6	59.38	25.73	95	258	53	39	46	856
2	21/F	Fever, cough	40.9	50	5.1	87.6	9.4	59.85	6.66	109	247	10	48	15	988
3	61/M	Fever, cough	40.8	35	8.2	83.8	11.0	76.12	1.22	114	235	18	87	35	561
4	32/M	Fever, cough, drowsiness, decreased breath sounds	40	50	5.16	71.0	23.6	46.7	5.77	118	162	23	169	161	3312
5	52/F	Fever, cough, drowsiness, decreased breath sounds, positive babinski sign	40.3	40	1.95	84.6	12.8	39.42	0.942	101	130	25	42	29	3043
6	13/M	Cough, fever, wheeze	39.8	60	3.04	33.5	57.7	5	3.32	100	90	171	44	128	4443
7	19/F	Fever, cough, wheeze, rash	39.9	48	0.51	9.8	80.4	166.75	25.91	86	66	15	31	49	1929
8	30/M	Fever, cough, wheeze	40	54	7.28	60.7	28.3	1.4	0.178	133	511	22	48	15	449

* *month; F, female; M, male; T_max_, maximum temperature; WBC, white blood cell count; CRP, C-reactive protein (normal range, <8 mg/L); PCT, procalcitonin (normal range, 0-0.46 ng/ml); ALT, alanine aminotransferase (normal range, <50 U/L); Cr, creatinine (normal range, 15-77 U/L); CK-MB, creatine kinase-MB (normal range, <25 U/L); LDH, lactate dehydrogenase (normal range, 110-295 U/L)*.

All 8 patients progressed to respiratory failure and complicated by ARDS in the early course of the disease. Arterial blood gas analysis taken before ECMO implementation revealed profound hypoxia with a median oxygen index of 23.75 (range, 5.0–52.6). Multiple organ failure was detected in 3 patients. Almost all the patients developed extrapulmonary manifestations, common in central nervous system (toxic encephalopathy in 4), digestive system (liver function lesion in 3; peptic ulcer, alimentary tract hemorrhage and pancreatitis in one, respectively), circulatory system (heart failure in 3, arrhythmia in 1), urinary system (acute renal failure in 2), hematologic system (anemia in 3, leukopenia in 3, thrombocytopenia in 4), and others including polyserous effusions in 8 and hypoproteinemia in 4 (Table 2).

**Table 2:** Radiographic manifestations, treatment and outcomes of patients with adenovirus pneumonia.

**No**	**Radiographic manifestations**	**OI**	Duration of dyspnea to IMV^*^	Duration of IMV^*^	**Duration of IMV to ECMO^†^**	Duration of ECMO^*^	**IVIG (g/kg)**	CRRT^*^	**Antiviral agent**	**Serotype**	**Coinfection**	Duration of ICU^*^	Duration of hospitalization^*^	**Main complications and sequelae**	**Outcomes**
1	Consolidations, pleural effusion	12.8	5	12	45.5	7	2	-	Oseltamivir	7	Negative	25	39	Plastic bronchitis, encephalatrophy	Survival
2	Consolidations, pleural effusion, atelectasis, bronchiectasis	18.9	5	13	22	9	2	-	Oseltamivir	NA	Influenza A virus, *Mycoplasma*	23	36	BO, hypertension	Survival
3	Consolidations, pleural effusion, bronchiectasis	28.6	7	15	24	8	4	-	Oseltamivir	NA	*Stenotrophomonas maltophilia*	42	77	BO, MOF, pancreatitis	Survival
4	Consolidations, pleural effusion, atelectasis	5.0	2	26	31	9	2.5	8	None	7	Negative	49	56	BO, MOF, pneumothorax, cognitive dysfunction	Survival
5	Consolidations, pleural effusion, bronchiectasis, pulmonary fibrosis	18.4	1	86	4	86	2	16	Acyclovir, oseltamivir	7	*Mycoplasma, Aspergillus fumigatus*	86	86	MOF, pneumothorax	Death (MOF)
6	Consolidations, pleural effusion, atelectasis, bronchiectasis	48.8	9	15	6	12	2.5	-	None	NA	*Klebsiella pneumoniae*	27	58	BO	Survival
7	Consolidations, pleural effusion, atelectasis	51.4	4	12	25	9	2	-	None	NA	Negative	28	28	BO, circuit clotting	Survival
8	Consolidations, pleural effusion, atelectasis	52.6	17	12	42	10	2	-	Oseltamivir	NA	*Mycoplasma, Acinetobacter baumannii*	12	28	Pneumorrhagia	Death (ARDS)

OI: oxygen index;

* day;

† *hour; IMV, invasive mechanical ventilation; ECMO, extracorporeal membrane oxygenation; IVIG, intravenous immunoglobin; CRRT, continuous renal replacement therapy; NA, not available; ICU, intensive care unit; BO, bronchiolitis obliterans; MOF, multiple organ failure; ARDS, acute respiratory distress syndrome*.

### Microbiological Data

Bronchoscopy with bronchial washings was performed in all 8 patients. HAdVs were detected in BALFs of 7 patients and nasopharyngeal swab sample of one patient by immunofluorescence assay. DNA of HAdVs was isolated in 3 patients (patient 1, 4, and 5) and genotyping revealed HAdV-7 for these 3 patients. Co-infections with *Mycoplasma, Stenotrophomonas maltophilia, Klebsiella pneumoniae, Acinetobacter baumannii, Aspergillus fumigatus* or influenza A virus were observed in 5 patients (Table 2).

### Radiographic Imaging

**Figure 1:**
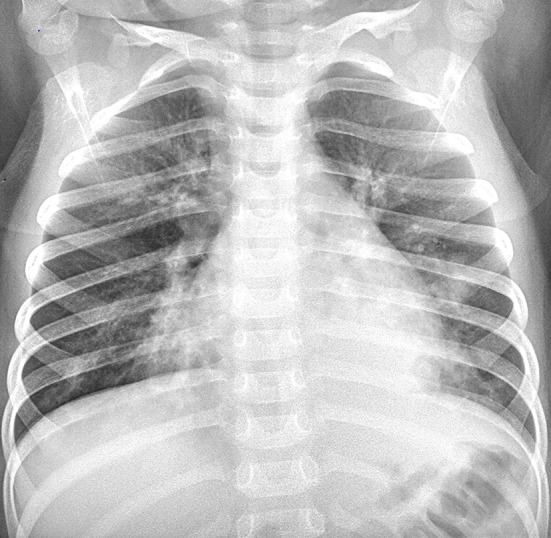
Dynamic changes on chest X-ray (CXR) and CT scans of our 2 patients. Patient 2: CXR showed bilateral patch ground glass opacities in both lungs on hospital day (HD) 1.

**Figure 2:**
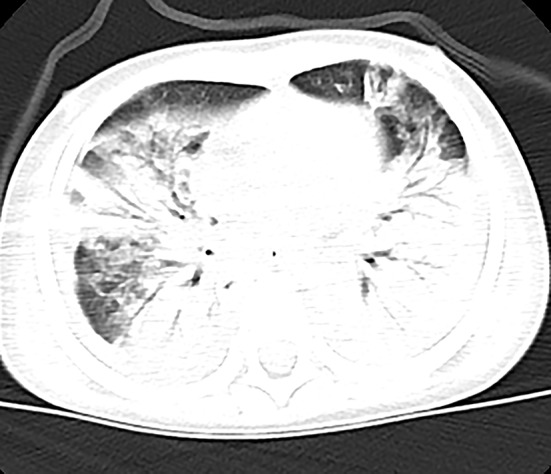
Patient 2: Bilateral pneumonic consolidations with air bronchogram and pleural effusion were found in CT on HD 6.

**Figure 3:**
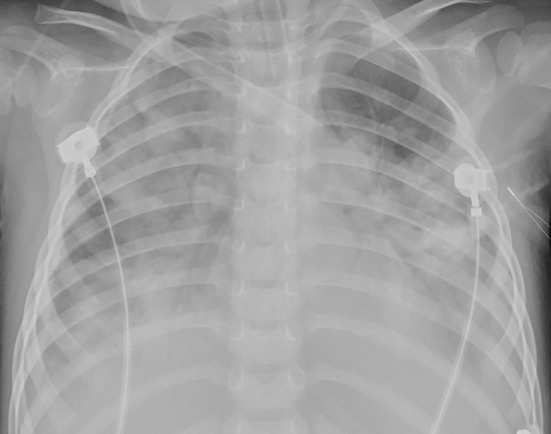
Patient 2: The consolidations aggravated and extracorporeal membrane oxygenation (ECMO) support was instituted on HD 7.

**Figure 4:**
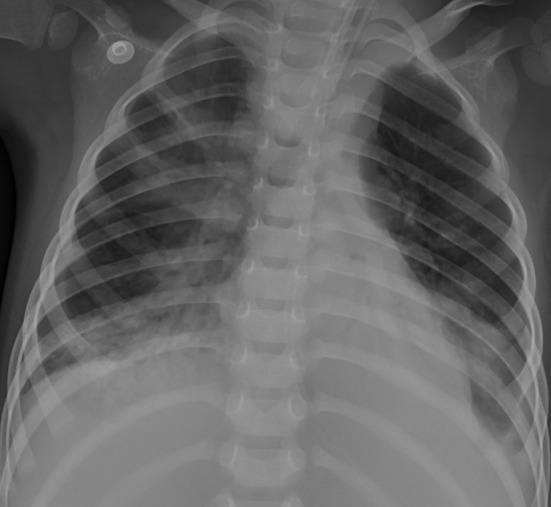
Patient 2: The pneumonic consolidations were improved on HD 16 after 9 days of ECMO therapy.

**Figure 5:**
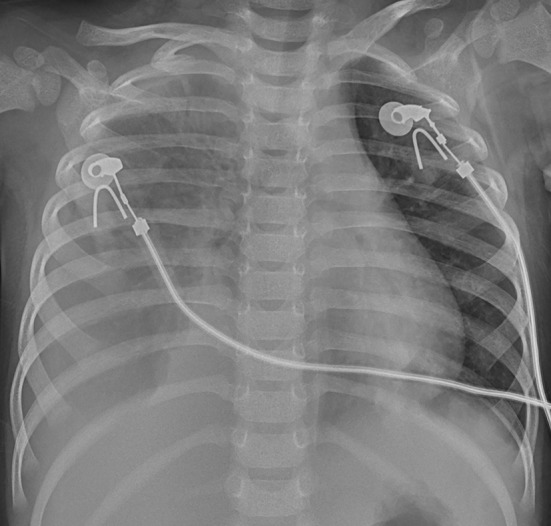
Patient 7: CXR taken on HD 1 showed pneumonic consolidations in the right lobe with right pleural effusion.

**Figure 6:**
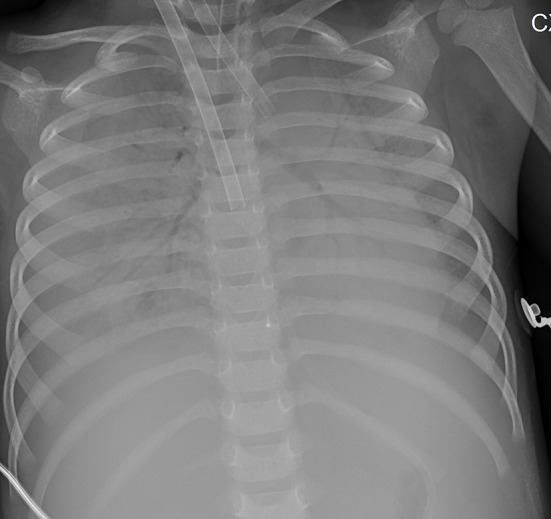
Patient 7: CXR revealed bilateral “white lung” like changes and obvious air bronchogram on HD 6, suggesting ARDS, and ECMO was implemented.

**Figure 7:**
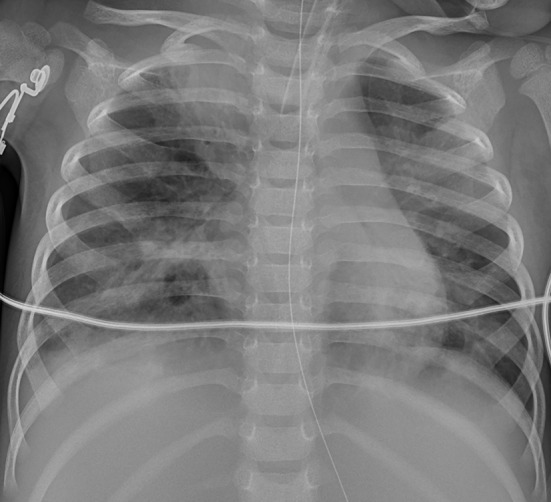
Patient 7: CXR showed improved pneumonic conditions with remaining consolidations and atelectasis of right upper lobe on HD 15 after 9 days of ECMO therapy.

**Figure 8:**
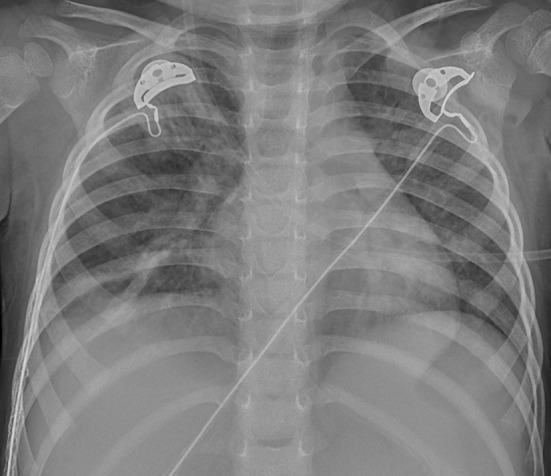
Patient 7: Consolidations in both lungs have further decreased on HD 29.

Chest X-ray and computed tomography (CT) scans revealed bilateral lobar or multifocal consolidations and air bronchogram in the lungs of all 8 patients, and radiographic lesions progressed rapidly after admission (Figures 1–8). Atelectasis and bronchiectasis were observed in 5 and 4 patients, respectively. Pulmonary fibrosis was identified in one patient. Pleural effusion was noted in all 8 patients. Consolidations and pleural effusion occurred almost simultaneously. The duration from onset of fever to development of pleural effusion ranged from 10 to 18 days with a mean duration of 15 days.

### Treatment and Outcome

Based on the seasonal outbreak and clinical and laboratory findings suggesting viral pneumonia, five patients were empirically treated with antiviral medications, including oseltamivir alone in 4 patients and oseltamivir combined with acyclovir in one patient. Empiric broad-spectrum antibiotics were initially administered but deescalated when microbiology results were available. Immunoglobulin was administered in the early course and periodically thereafter, in total doses varying between 2 and 4 g/kg. All the patients received steroid therapy. Continuous renal replacement therapy treatment was instituted in 2 patients (patient 4 and 5) for acute renal failure (Table 2).

The median duration prior to development of respiratory failure requiring intubation and invasive mechanical ventilation was 5 days. Venoarterial ECMO supports as rescue ventilation was instituted after the median time of 24.5 h of conventional mechanical ventilator support. The median duration on ECMO support and mechanical ventilation were 9 and 14 days, respectively. Median length of stay in ICU and hospital were 27.5 and 47.5 days, respectively (Table 2). The oxygenation response to ECMO was shown in Table 3.

**Table 3:** Oxygenation response to extracorporeal membrane oxygenation support.

**No**	**ECMO model**	**Mechanical ventilation settings pre-ECMO**			**Pre-ECMO arterial blood gas analysis**				**Mechanical ventilation settings post ECMO weaning**			**Post ECMO weaning arterial blood gas analysis**			
		**Ventilator type**	**PEEP (cmH_**2**_O)**	**FiO_**2**_ (%)**	**pH**	**PaO_**2**_ (mmHg)**	**PaCO_**2**_ (mmHg)**	**SpO_**2**_ (%)**	**Ventilator type**	**PEEP (cmH_**2**_O)**	**FiO_**2**_ (%)**	**pH**	**PaO_**2**_ (mmHg)**	**PaCO_**2**_ (mmHg)**	**SpO_**2**_ (%)**
1	Medos 2400	SIMV	3	1	7.256	46.9	66.8	83	SIMV	7	0.55	7.256	92.9	58.3	97
2	Medos 2400	SIMV	13	1	7.473	58.2	41.2	78	SIMV	8	0.5	7.386	129	46.7	100
3	Sorin	SIMV	8	0.98	7.471	48	47.1	86	SIMV	6	0.8	7.329	99.2	46.1	97
4	Medos 2400	HFOV	f =6 Hz ΔP = 40 MAP = 9	0.4	7.204	71.3	61.3	84	SIMV	6	0.7	7.525	153	33.2	99
5	Sorin	APRV	P_high_ = 29 P_low_ = 7	1	7.332	43.5	44.4	59	-	-	-	-	-	-	-
6	Medos 2400	APRV	P_high_ = 30 P_low_ = 7	1	7.383	43	41.8	69	SIMV	6	0.4	7.457	157	42.4	100
7	Sorin	APRV	P_high_ = 34 P_low_ = 2	1	7.498	50.6	33.9	80	SIMV	7	0.4	7.36	114	44.6	100
8	Medos 2400	HFOV	f = 5 Hz ΔP = 48 MAP = 23	0.99	7.226	43.3	83.6	40	-	-	-	-	-	-	-

*ECMO, extracorporeal membrane oxygenation; SIMV, synchronized intermittent mechanical ventilation; HFOV, high-frequency oscillatory ventilation; APRV, airway pressure release ventilation; PEEP, positive end-expiratory pressure; MAP, mean airway pressure; FiO_2_, fraction of inspired oxygen; PaO_2_, partial pressure of oxygen; PaCO_2_, partial pressure of carbon dioxide; SpO_2_, peripheral capillary oxygen saturation*.

Some complications, including bleeding, infection, pneumothorax, circuit clotting, hypertension, neurological dysfunction and acute kidney injury, were noted during ECMO support (Table 2). Unfortunately, two patients (25.0%) died. A 52-month-old girl (patient 5) received 86 days of ECMO and mechanical ventilator support, but pulmonary imaging showed progressive exacerbation of pulmonary fibrosis. Although lung transplantation was conducted on the day 78 of ECMO therapy, she eventually died of multi organ failure. Another 30-month-old boy (patient 8) received 10 days of ECMO support and 12 days of mechanical ventilator support. However, he manifested coagulation dysfunction together with progressively decreasing oxygen saturation. Finally, his parents gave up all the treatments, and the patients passed away after staying in hospital for 28 days (Table 2).

Duration of follow-up was 7-22 months for the 6 survivors. Recurrent cough or wheezing was infrequent. BO was observed in 5 of the 6 survivors. Plastic bronchitis was found by flexible bronchoscopy and confirmed by pathological examination in patient 1. Other residual pulmonary diseases, such as atelectasis, necrotizing pneumonia and bronchiectasis, were found in one, one and 3 patients, respectively.

### Risk Factors Analysis

The demographic and clinical features of the severe adenovirus pneumonia group were compared with those in the common adenovirus pneumonia group (Table 4). Severe adenovirus pneumonia group showed older age (*p* = 0.005), lower white blood cell levels (*p* = 0.008), higher C-reactive protein (*p* = 0.022), procalcitonin (*p* = 0.001), and lactate dehydrogenase (*p* = 0.001) levels. Pleural effusion (*p* < 0.001) was seen more frequently in the severe adenovirus pneumonia group. There is no difference of sex or maximum body temperature between these two groups.

**Table 4:** Comparisons of demographic and clinical features with the two groups.

**Variables**	**Severe adenovirus pneumonia (*n* = 8)**	**Common adenovirus pneumonia (*n* = 100)**	***p*-value**
Age (months)	31.0 (19.5-51.3)	19.0 (7.0-43.5)	0.005
Male (%)	4/8 (50.0)	61/100 (61.0)	0.813
T_max_ (°C)	40.0 (39.9-40.7)	39.8 (39.1-40.2)	0.072
WBC (10^9^/L)	5.1 (2.2-8.0)	10.1 (6.6-13.5)	0.008
Neutrophil (%)	77.4 (40.3-85.2)	54.4 (30.9-68.0)	0.105
CRP (mg/L)	53.0 (13.6-72.1)	8.7 (1.7-25.4)	0.022
PCT (ng/ml)	4.5 (1.0-21.0)	0.3 (0.1-0.6)	0.001
LDH (U/L)	1458.5 (634.8-3244.8)	408.0 (282.0-639.0)	0.001
Pleural effusion (%)	8/8 (100.0)	13/100 (13.0)	<0.001

*T_max_, maximum temperature; WBC, white blood cell count; CRP, C-reactive protein (normal range, <8 mg/L); PCT, Procalcitonin (normal range, 0-0.46 ng/ml); LDH, lactate dehydrogenase (normal range, 110-295 U/L)*.

## Discussion

We reported 8 immunocompetent patients of severe adenovirus pneumonia associated with ARDS in whom ECMO support was required, and showed a promising survival rate of 75%. Furthermore, to our knowledge, this is the first retrospective observational study on the comparison of clinical manifestations between severe adenovirus pneumonia requiring ECMO support and common adenovirus pneumonia. In this regard, several findings are noteworthy. (1) HAdV could cause severe adenovirus pneumonia accompanied by ARDS in immunocompetent patients in whom conventional mechanical ventilation failed and ECMO support was required. (2) Older age, elevated levels of C-reactive protein, procalcitonin and lactate dehydrogenase, and rapid development of bilateral consolidations and pleural effusions may be the risk factors of severe adenovirus pneumonia, which may induce ARDS and required ECMO support. (3) ECMO may be considered as the last support method when advanced life support steps were performed without clinical response. (4) The promising outcome in our group may be partly explained by the early institution of ECMO and the appropriate duration of ECMO support.

All patients in our study were from the same region, Zhejiang province, southern China, implying another outbreak of life-threating adenovirus pneumonia in South China in the decade (11). The 8 cases of severe adenovirus pneumonia occurred in December and between February and July, highly similar to the previous reports that epidemics of adenovirus respiratory disease were common during the winter, spring, and early summer (29, 30).

The clinical course of adenovirus in immunocompetent patients is usually benign and most patients recover spontaneously, and reports on life-threatening adenovirus pneumonia in previously healthy patients are not common (1, 10). Our patients were all previously healthy children without underlying disease. Our results suggested that the followings may be the typical manifestations of severe adenovirus pneumonia: cough and persistent ardent fever up to 40, rapid progression to respiratory failure within 1 week, respiratory compromise with hypoxia and rapid deterioration of clinical condition, requiring intubation and mechanical ventilation. Additionally, lymphopenia, thrombocytopenia, and elevated C-reactive protein, procalcitonin, and lactate dehydrogenase levels are frequently observed. The rapid development of bilateral consolidations was the most common radiographic finding, usually accompanied by adjacent ground glass opacities and pleural effusions. The clinical features were highly similar to those of severe adenovirus infections described in previous reports (10, 11, 31, 32). Since almost all the 8 patients developed multiple organ function impairment, it was likely that patients in our cohort had disseminated adenovirus disease, an entity defined as the presence of systemic adenoviral infection involving two or more organs. A retrospective review of adenovirus infections reported that disseminated adenovirus disease with multiorgan involvement occurred in 11 of 440 (2.5%) adenovirus infected patients with a high mortality of 60% even in immunocompetent host (33). This indicated the severity and lethality of severe adenovirus pneumonia.

Though positive medical therapies and mechanical ventilator supports were performed on these 8 patients, their conditions continued to deteriorate. It may be associated with the insult of HAdV to the lower respiratory tract, resulting in BO, pulmonary fibrosis and other sequelae, the occurrence of ARDS and the disseminated adenovirus diseases. By comparisons with clinical features of common adenovirus pneumonia group, we noted that older age, noticeable raised levels of C-reactive protein, procalcitonin, lactate dehydrogenase, and the frequent presence of pleural effusions might be the risk factors of severe adenovirus pneumonia, which required ECMO support.

More and more researches supported that consolidations rather than interstitial infiltrates as the main radiological findings of adenovirus pneumonia. In our study, bilateral lobar or multifocal consolidations occurred in the lungs of all the 8 patients with severe adenovirus pneumonia. To date, adenovirus is the only virus known to cause focal or lobar consolidation as its main imaging characteristic (32), which resembles typical radiographic manifestation of bacterial pneumonia, and may mislead diagnosis. Nonetheless, the presence of wheeze, the diffuse nature of auscultatory abnormalities which were not confined to areas of consolidation, normal or decreased white blood cell counts, findings that include bilateral and multifocal involvement on chest radiographs, and progression of illness despite extensive antibiotic therapy, help to differentiate adenoviral from bacterial pneumonia (15, 34).

It has been noted that 14–60% of children with documented lower respiratory disease due to adenovirus have some degree of pulmonary sequelae (21, 35, 36). Compared with other viruses responsible for lower respiratory tract infections in childhood, adenovirus causes more severe respiratory and extra pulmonary manifestations (37) and is more prone to be followed by the development of BO (38). All the 6 survivors in our study have developed pulmonary sequelae, and BO (83.3%) was the most common manifestation. Plastic bronchitis was found in one patient, providing the rare case of HAdV infection-induced plastic bronchitis. Pulmonary fibrosis was observed in another patient, indicating the poor prognosis when it occurred in patients with adenovirus pneumonia.

Previous data analysis showed that for children requiring ECMO support for adenovirus infection, the survival rate was only 25% (39), the lowest among the viral types (11, 13, 31, 40), while another review of the Extracorporeal Life Support Organization Registry published in 2014 showed the survival rate at hospital discharge was 38% (41). It was demonstrated that early institution of ECMO in a ventilated patient was associated with improved survival, and late cannulation beyond 6 days was related to an increase in mortality (42, 43). Besides, Prolonged duration of ECMO support was supposed to be a risk factor of increased mortality (41, 42), which may be partly attributed to a great number of ECMO-related complications and the severity of the disease itself. In our group, the median duration of mechanical ventilation prior to initiation of ECMO was only 24.5 h. Although the longest duration of ECMO support was up to 86 days, the patient eventually died due to multiple organ failure, and the median duration on ECMO support was 9 days. The survival rate (75%) of our 8 patients of severe adenovirus pneumonia associated with ARDS was higher compared to that in previous studies, which might be partly explained by the early institution of ECMO and the appropriate duration of ECMO support.

A number of complications occurred in our patients during the ECMO support, including bleeding, infection, pneumothorax, circuit clotting, hypertension, neurological dysfunction, and acute kidney injury, consistent well with the findings in the previous studies (41, 43, 44), and may represent the increased risk of maldistribution of oxygenated blood and systemic thromboembolism in patients supported on venoarterial ECMO (45). Venoarterial ECMO was demonstrated to be associated with more serious complications, e.g., cerebral infarction, seizures, and a higher mortality than venovenous ECMO (42, 45, 46). Nevertheless, because of the hemodynamic instability and/or cardiovascular dysfunction, the 8 patients with severe adenovirus pneumonia had to be placed on venoarterial ECMO. It was reasonable to hypothesize that the outcomes of these patients could potentially be better at institution with venovenous ECMO. Acute kidney injury was frequently present at the initiation of ECMO and had a significant association with increased duration of ECMO support and increased risk of mortality (47). In this context, concurrent continuous renal replacement therapy with ECMO was technically feasible and efficacious in the management of fluid overload and solute clearance (48). The use of antiviral agents to treat adenovirus is not well established. Cidofovir was supposed to be a therapeutic option in adenoviral ARDS (49), but its benefit had to be weighed against increased nephrotoxicity, and significant toxicity has so far limited its use more broadly (50). Actually, cidofovir is not available in most hospitals in China (51), including our hospital. The mainstay of treatment for patients with severe adenovirus pneumonia is still supportive, and ECMO would be applied when pediatric advanced life support steps are performed thoroughly without clinical response.

Our study has several limitations. First, our study was a single-center and retrospective study, and had a selection bias. Second, specific serotyping of adenovirus from our 5 cases was not available. Lastly, the number of cases in the present study is small, and a large-scale prospective study is urgently needed.

## Conclusion

Our results showed that HAdVs could cause severe adenovirus pneumonia accompanied by ARDS in immunocompetent patients. Older age, markedly elevated levels of C-reactive protein, procalcitonin, and lactate dehydrogenase may be the risk factors of severe adenovirus pneumonia requiring ECMO support. The promising outcome in our patients suggested that ECMO support should be instituted in the course of respiratory failure in children with severe adenovirus pneumonia, and may improve outcome.

## Data Availability Statement

All datasets generated for this study are included in the article/supplementary material.

## Ethics Statement

The study has been approved by the Medical Ethical Committee of the Children's Hospital of Zhejiang University School of Medicine (Hangzhou, China). The study does not contain patients' personal information, fully protects the patients' privacy, has no effects on the routine diagnosis and treatment of patients, patients do not participate in the study for additional tests or examinations. Written informed consents from the participants' legal guardian/next of kin was not required to participate in this study in accordance with the national legislation and the institutional requirements.

## Author Contributions

XC analyzed the data and wrote the manuscript. JL, LQ, and CZ collected the data and followed up the prognosis. LT performed the study design and critical revision. All authors read and approved the final manuscript and agreed to be accountable for the content of the work.

### Conflict of Interest

The authors declare that the research was conducted in the absence of any commercial or financial relationships that could be construed as a potential conflict of interest.
